# Clinical value of styrofoam fixation in intracranial tumor radiotherapy

**DOI:** 10.3389/fonc.2023.1131006

**Published:** 2023-03-27

**Authors:** Bo Li, Fei Bai, Xiaowei Yao, Linlin Xu, Lina Zhao

**Affiliations:** Department of Radiation Oncology, Xijing Hospital, Fourth Military Medical University, Xi’an, China

**Keywords:** intracranial tumor, CBCT, setup error, radiation, styrofoam

## Abstract

**Objective:**

To analyze the application value of two postural fixation techniques.(styrofoam combined with head mask and fixed headrest combined with head mask) in intracranial tumor radiotherapy *via* cone beam computed tomography (CBCT).

**Methods:**

This study included 104 patients with intracranial tumors undergoing radiotherapy. The patients were divided into two groups: Group A (54 cases with styrofoam fixation) and Group B (50 cases with fixed headrest fixation). The positional deviation in 3D space between the two groups was compared using CBCT. The set-up errors were expressed as median (25th percentile, 75th percentile)or M(p25, p75) since the set-up errors in all directions were not normally distributed,The Mann-Whitney U test was performed.

**Results:**

The age and gender of patients in the two groups were not significantly different. The set-up errors of A in lateral (X), longitudinal (Y), vertical (Z), and yaw(Rtn) axes were 1.0 (0,1) mm, 1.0 (0,1) mm, 1.0 (0,2) mm, and 0.4 (0.1, 0.8) degrees, respectively while the set-up errors of B were 1.0 (0,1) mm, 1.0 (1,2) mm, 1.0 (0,2) mm, and 0.5 (0.15,0.9) degrees, respectively. Moreover, patients in the styrofoam group had significantly smaller set-up errors in the Y-axis than patients in the headrest group (p=0.001). However, set-up errors in the X, Z, and Rtn axes were not significantly different between the two groups. The expansion boundaries of the target area in the X, Y, and Z directions were 1.77 mm, 2.45 mm, and 2.47 mm, respectively. The outer expansion boundaries of the headrest group were 2.03 mm, 3.88 mm, and 2.57 mm in X, Y, and Z directions, respectively. The set-up times of groups A and B were (32.71 ± 5.21) seconds and (46.57 ± 6.68) seconds, respectively (p=0.014). Patients in group A had significantly better comfort satisfaction than patients in group B (p=0.001).

**Conclusion:**

Styrofoam plus head thermoplastic mask body fixation technique has a higher positional accuracy in intracranial tumor radiotherapy than headrest plus head thermoplastic mask fixation. Besides, styrofoam plus head thermoplastic mask body fixation technique is associated with improved positioning efficiency, and better comfort than headrest plus head thermoplastic mask fixation, and thus can be effectively applied for intracranial tumor radiotherapy positioning.

## 1 Introduction

Primary intracranial tumors originate from the neuroepithelium, meninges, cranial nerves, and brain tissue, while secondary intracranial tumors are formed in the skull *via* metastases of other tissues or organs. Radiation therapy (RT), such as stereotactic radiosurgery (SRS), conventionally fractionated radiotherapy, and stereotactic radiotherapy (SRT), can be used to treat intracranial tumors. Unlike traditional RT, SRS can deliver high doses of radiation in a single treatment session. SRT refers to a similar stereotactic approach for approximately 2 to 5 treatments (1–4), indicating that a higher target area precision is needed during SRT for intracranial tumor radiotherapy to improve the accuracy and reproducibility of the radiotherapy body position (5).

Although fixed headrest with a head mask is the conventional postural fixation for intracranial tumor radiotherapy, it can shrink and deform over time, thus affecting the positional accuracy of patient treatment. However, personalized custom headrests can avoid this problem and improve radiotherapy accuracy (6). Ghazal Shafai-Erfani et al. (7) found systematic errors of 1.1, 2.0, and 2.3 mm in the left-right, head-foot, and anterior-posterior directions, respectively, for the headrest plus mask fixation technique. M. Mattke et al. (8) found mean positional errors of 0.9, 2.28, and 1.9 mm in the left-right, head-foot, and anterior-posterior directions, respectively, for patients undergoing whole-brain radiotherapy using headrest plus mask fixation. These findings indicate that the accuracy of standard headrest and mask fixation should be improved.

Veronika M. Miron et al. (9) fabricated a new comfortable positioning head mask *via* a 3D printing technique with higher fixation accuracy. M. Mattke et al. (8) also found the fixation accuracy of 3D-printed cephalic masks is lower than that of conventional cephalic fixation. Nonetheless, the fixation accuracy of 3D-printed cephalic masks is within the allowable error range. Although 3D printing technology can increase patient comfort, the high technical requirements limit its scaling.

Xu Senkui et al. (10) also showed that the set-up error of styrofoam is smaller than that of standard headrest fixation techniques, especially in the neck. Lin Chengguang et al. (11) also showed that the styrofoam fixation technique has a better positional accuracy both in the nasopharyngeal and cervical regions than the standard headrest and vacuum pad. Foamed gel is a common positioning technique used for radiotherapy of head and neck tumors, but its effect in intracranial tumors is unknown.

This study aimed to provide a more accurate and comfortable fixation technique for patients undergoing intracranial tumor radiotherapy by analyzing and comparing the positional errors of both fixation techniques and evaluating their comfort using a radiotherapy fixation device comfort questionnaire.

## 2 Materials and methods

### 2.1 Patients and data collection

This study included 104 patients with intracranial tumors who received radiotherapy in the Department of Radiotherapy of our hospital from December 2021 to August 2022 (54 cases were fixed with Styrofoam and 50 were fixed with headrests). The demographic and general clinical data of the patients are shown in **Table 1**. This study was approved by the hospital’s Research, and Ethics committee (KY20212133-F-1)and all patients signed an informed consent form.

**Table 1:** General clinical data of the patients.

Characteristic		Styrofoam group	Fixed headrest group
Number of patients		54	50
Age (years) (range)		51 ± 13(26-89)	51 ± 12(15-70)
Sex	male	31	33
	female	23	17
Operation	Post operation	39	33
	radical operation	15	17
Tumor type	Glioma of brain	34	30
	Brain metastasis	13	17
	Cerebral angioma	2	0
	Cerebral lymphoma	2	0
	Meningioma	3	2
	Not specified	0	1

### 2.2 Fixation position

**Figure 1:**
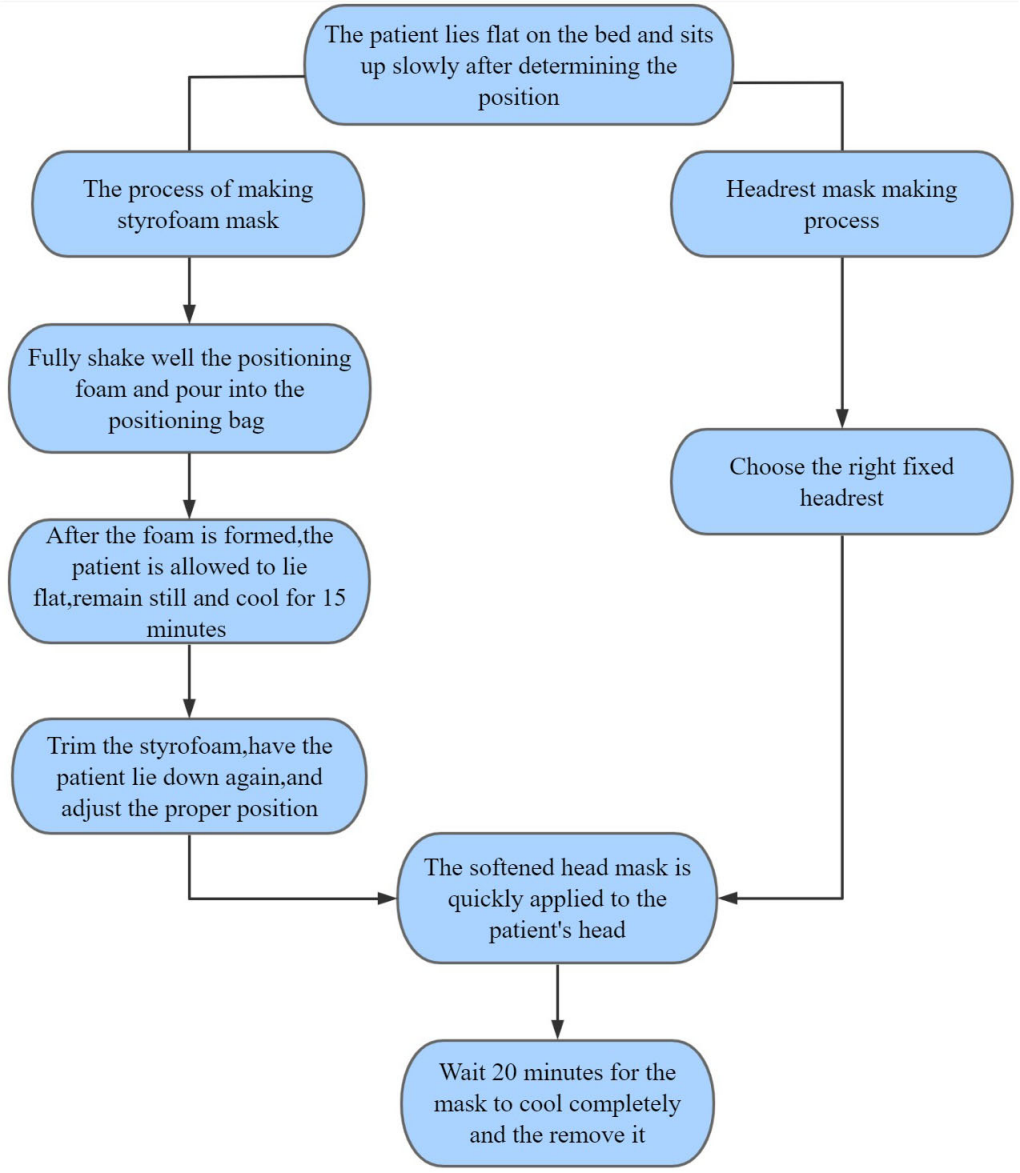
Flowchart.

The production of head thermoplastic mask and styrofoam is shown in the flow chart in **Figure 1**. The positioning head frame and bag were placed on the carbon one-piece plate as the patient laid flat on the carbon plate with the body mid-sagittal plane perpendicular to the couch surface. The patient slowly sat up after determining the position, and kept still. The positioning technician thoroughly shook the positioning foam and poured it evenly into the positioning bag. The patient then lay down and kept still, and the bag was sealed quickly. The positioning foam pad was allowed to cool for 15 min, then the patient sat up, and the positioning foam pad was cut and trimmed. The patient then laid down again, and the head position was adjusted under the laser light to ensure the orbital ear line on both sides was perpendicular to the couch surface. The patient was asked not to move, and the Klarity head mask was placed in a constant temperature water tank at 65 °C-75 °C. The mask was transparently softened (about 3 min), then put on the patient’s head. The three technicians stuck the mask according to the patient’s outline. The mask was completely cooled after 20 min, finalized, and removed.

**Figure 2:**
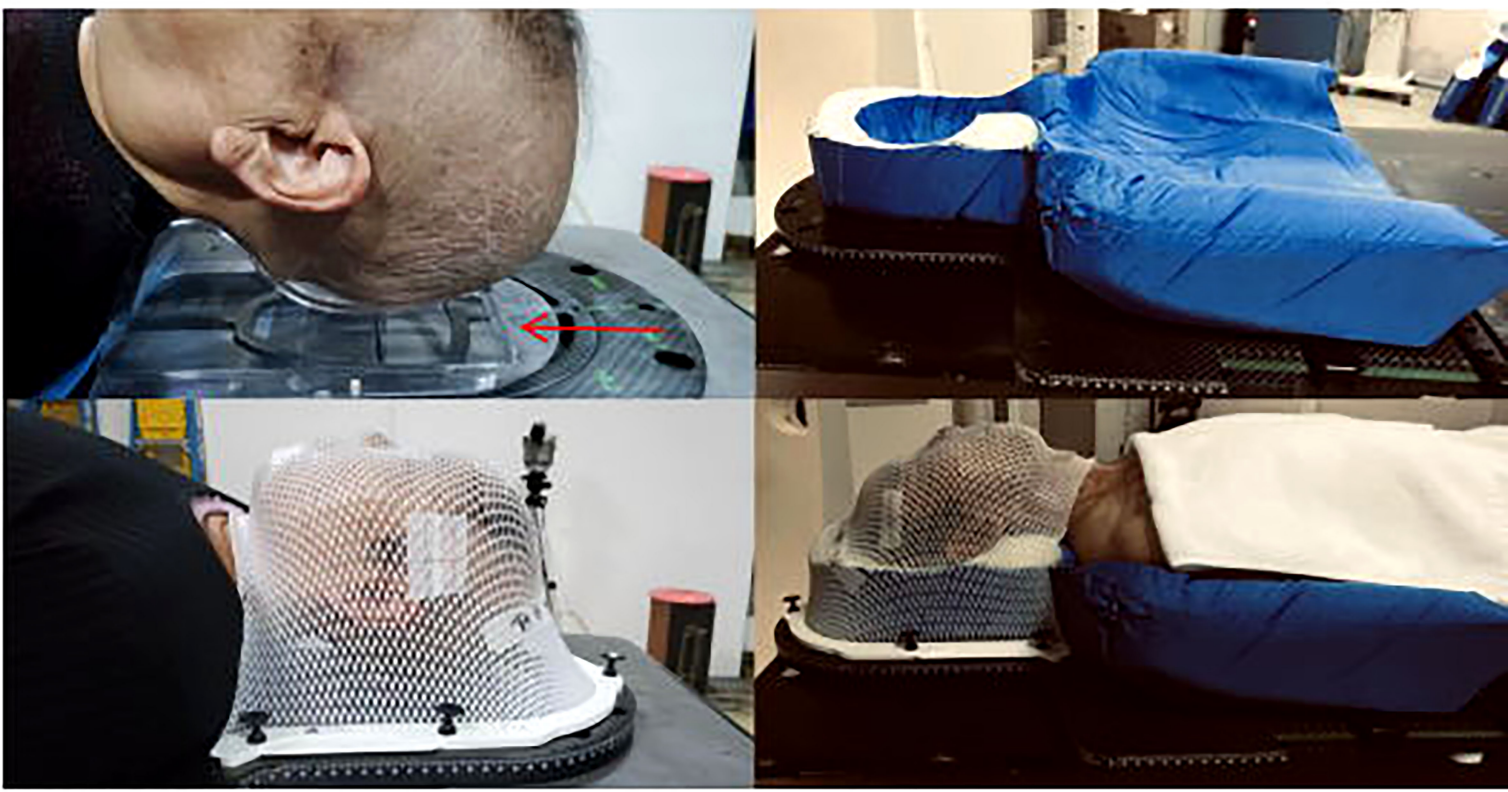
Fixation technique:fixed headrest technique (left) and styrofoam fixing technique (right).

The head thermoplastic mask with headrest was made by selecting the appropriate headrest from five types of headrests (A, B, C, D, and F) according to the patient’s head and neck fit and the patient’s self-reported comfort level (The headrest is made of plastic (suppliers:Klarity).). This was conducted by placing the headrest on the carbon plate and asking the patient to lie flat on the carbon plate with the mid-sagittal plane of the body perpendicular to the couch.then the head thermoplastic mask was made, as shown in **Figure 2**.

### 2.3 Image acquisition registration and error data collection

The patients were fixed using the two methods. The Philips Big Bore CT simulator was then used for plain and enhanced scans. The obtained images were used as the original reference images for posterior set-up verification, while the CT scan images were transferred to Varian Eclipse 8. 9 planning system through the network. The gross tumor volume (GTV), clinical target volume (CTV), planning target volume (PTV) range, and the expansion range of the target volume were defined according to the ICRU50 and ICRU62 documents combined with the actual situation of the department, then delineated at the corresponding level. Different colors were used to represent different target areas. The radiation therapy physicist developed the individualized volumetric rotary intensity-modulated radiotherapy (VMAT) plan according to the requirements of the plan request form. The body position was fixed under the guidance of the laser locator in the accelerator room according to the parameters of the treatment plan, and the fixation was consistent with that in CT. Scanning was performed using the CBCT on the Varian Clinac ix linear accelerator. The 3D image of the treatment site was also obtained. After acquiring the images automatically matched by the machine, the attending physician and technician need to confirm the accuracy of the target area (and adjust the position of the images, if necessary). The translation vector and rotation degree in the directions were also recorded. The patients were scanned using kV-CBCT before the first treatment and compared with the planned CT images to obtain the spatial position deviation of the two images (error value of the target center in X, Y, and Z directions and rotation). Detailed records were made after the physician confirmed the images. A correction was made if there was a deviation of more than 3 mm in each direction (re-positioning). Furthermore, the isocenter was corrected if the error was more than 3 mm twice. The couch was moved directly if the error was less than 3 mm. The doctor then decided the number of CBCT verification according to the fraction dose of the patient (fraction dose ≤3Gy, every 5 treatment verifications performed once; fraction dose > 3Gy and < 5Gy, every two treatment verifications performed once (watched by technicians online and by doctors offline). The error value was also recorded during the above process. Verification was required before each treatment if the fraction dose of treatment was ≥5Gy (watched online by technicians and physicians). The error values were also recorded. A CBCT scan was performed at any time when mask factors (mask treatment marker line missing) or patient factors (changes in head position due to patient weight gain or loss) affected the accuracy of treatment. The set-up error of each patient was calculated as described by Stroom et al. (12). The data errors came from two aspects: systematic errors and random errors. The systematic error Σ (expressed as the average value of all set-up errors) was mainly derived from the mechanical parameter errors of the machine and equipment during positioning and treatment while the random error δ (expressed as the standard deviation of all set-up errors) was mainly the difference in repeatability of each set-up during radiotherapy (13). The expansion of CTV to PTV in X, Y, and Z directions was calculated using the formula described by Vanherk (14) (M_PTV_Σ2.5+0.7δ). The outer extension range was calculated based on the expansion of CTV to PTV in X, Y, and Z directions.

### 2.4 Subjective comfort

A questionnaire survey was conducted for the first treatment, mainly to understand patients’ subjective comfort satisfaction with the device. The satisfaction survey comprised eight items, each with a 5-point Likert scale. The dimensions of evaluation were: head, neck, and back comfort, mask fit, tightness, temperature, color, anxiety about the fixture, general discomfort, and whether the fixture was recommended.

### 2.5 Statistical analysis

Statistical analysis was conducted using SPSS25.0 statistical software. Enumeration data were expressed as frequency (n) and percentage (%). Differences between groups were compared using χ2 test. The set-up errors were expressed as median (25th percentile, 75th percentile) or M(p25, p75) since the set-up errors in all directions were not normally distributed. Mann-Whitney U test was also conducted. P< 0.05 was considered statistically significant.

## 3 Results

### 3.1 General patient data

A total of 537 valid CBCT images were acquired from all patients. A total of 278 CBCT images were acquired in the styrofoam group, with a re-positioning correction rate of 10.4% (29/278) and a first treatment re-positioning correction rate of 11.1% (6/54). A total of 259 valid images were acquired in the headrest group with a re-positioning correction rate of 16.6% (43/259) and a first treatment re-positioning correction rate of 20.0% (10/50). The overall re-positioning correction rate was significantly different between the two groups (χ2 = 4.397, P=0.036), while the first treatment re-positioning correction rate was not significantly different (χ2 = 1.576, P=0.209).

### 3.2 Overall patient positional errors

**Figure 3:**
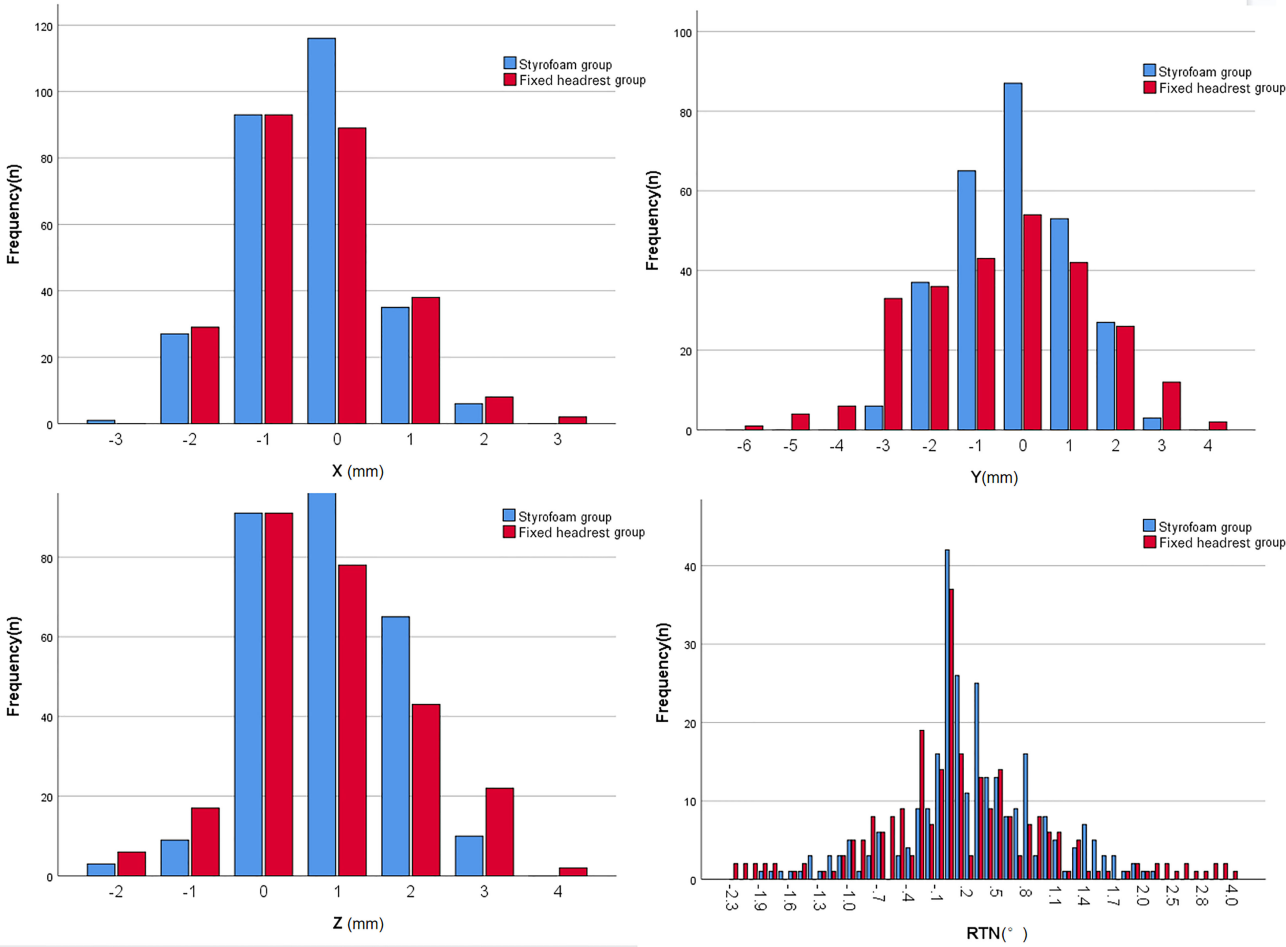
Histogram of the overall range of positional error in the two groups.

The error ranges of patients in both groups are shown in **Figure 3**. The errors in the X, Y, Z, and RTN axes were 1.0 (0, 1) mm, 1.0 (0, 1) mm, 1.0 (0, 2) mm, and 0.4 (0.1, 0.8)°, respectively, for the styrofoam group and 1.0 (0, 1) mm, 1.0 (1, 2) mm, 1.0 (0, 2) mm, and 0.5 (0.1, 0.9)° for the headrest group, respectively. The positional errors in the Y direction were significantly different between the two groups (Z = 5.457, P < 0.001) and not significantly different in the X, Z, and RTN directions (P > 0.05). The detailed data are shown in **Table 2**.

**Table 2:** Comparison of the overall set-up error between the two groups.

Group	Number of CBCT	Setup errors(mm)			
		X	Y	Z	RTN(°)
Styrofoam group	278	1(0,1)	1(0,1)	1(0,2)	0.4(0.1,0.8)
Fixed headrest group	259	1(0,1)	1(1,2)	1(0,2)	0.5(0.1,0.9)
Z value		1.793	5.457	0.006	1.750
P		0.073	<0.001	0.995	0.080

### 3.3 Postoperative set-up errors

**Figure 4:**
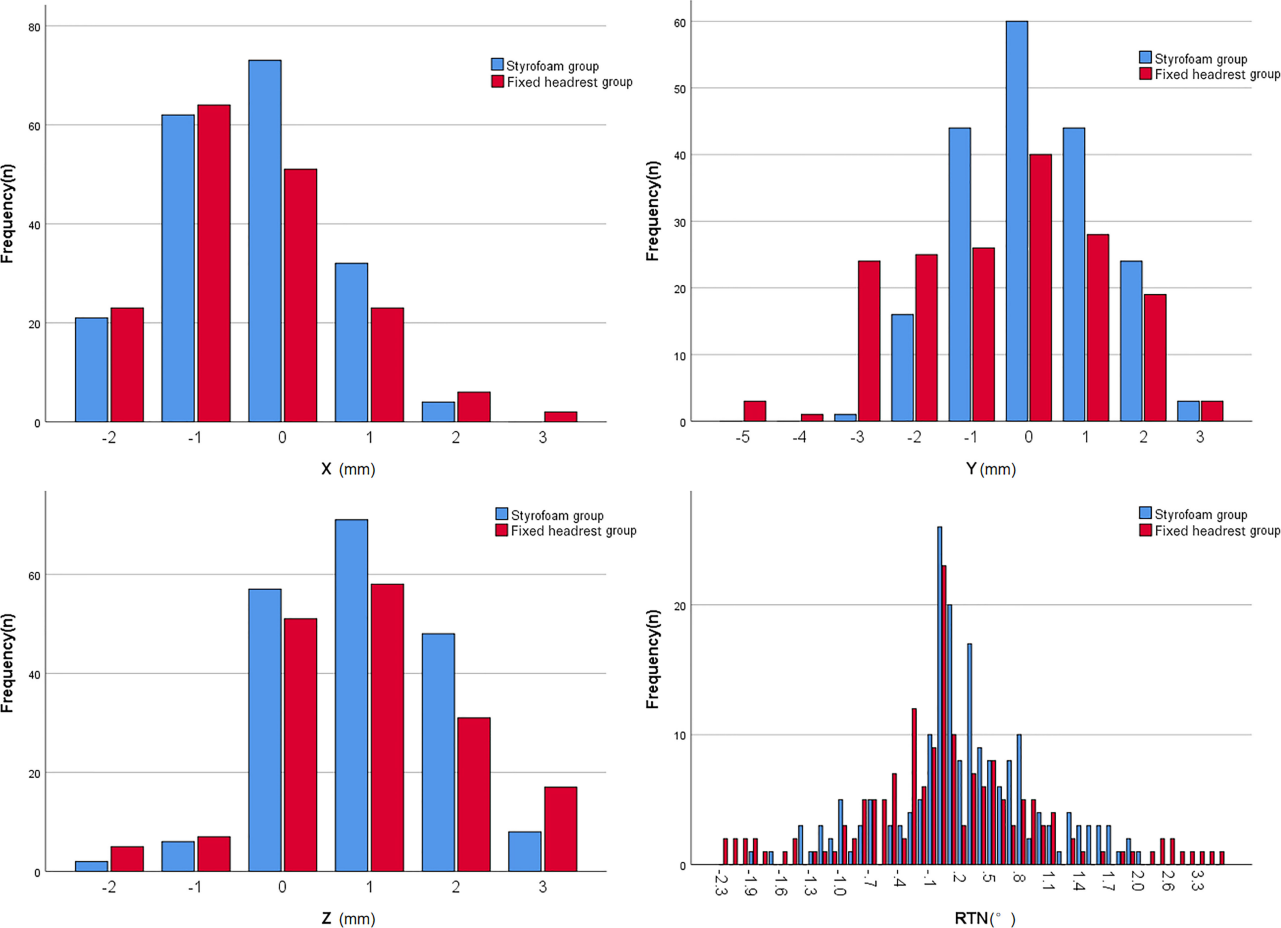
Histogram of the range of set-up error in the two postoperative groups.

The error ranges in the two groups are shown in **Figure 4**. The errors in the X, Y, Z, and RTN axes were 1.0 (0, 1) mm, 1.0 (0, 1) mm, 1.0 (0, 2) mm, and 0.4 (0.1, 0.8)°, respectively, for the foam gel group and 1.0 (0, 1) mm, 1.0 (1, 2) mm, 1.0 (0, 2) mm, and 0.5 (0.15, 0.9)°, respectively, for the headrest group. The positional errors in the Y-axis were significantly different between the two groups (Z=4.125, P<0.001) and not significantly different in the X, Z, and RTN directions (P>0.05). The detailed data are shown in **Table 3**.

**Table 3:** Comparison of set-up errors between two groups of patients undergoing postoperative radiotherapy.

Group	Number of CBCT	Setup errors(mm)			
		X	Y	Z	RTN(°)
Styrofoam group	192	1(0,1)	1(0,1)	1(0,2)	0.4(0.1,0.8)
Fixed headrest group	169	1(0,1)	1(1,2)	1(0,2)	0.5(0.15,0.9)
Z value		1.710	4.125	0.381	0.634
P		0.087	<0.001	0.703	0.526

### 3.4 Positional errors in patients treated with radical radiotherapy

**Figure 5:**
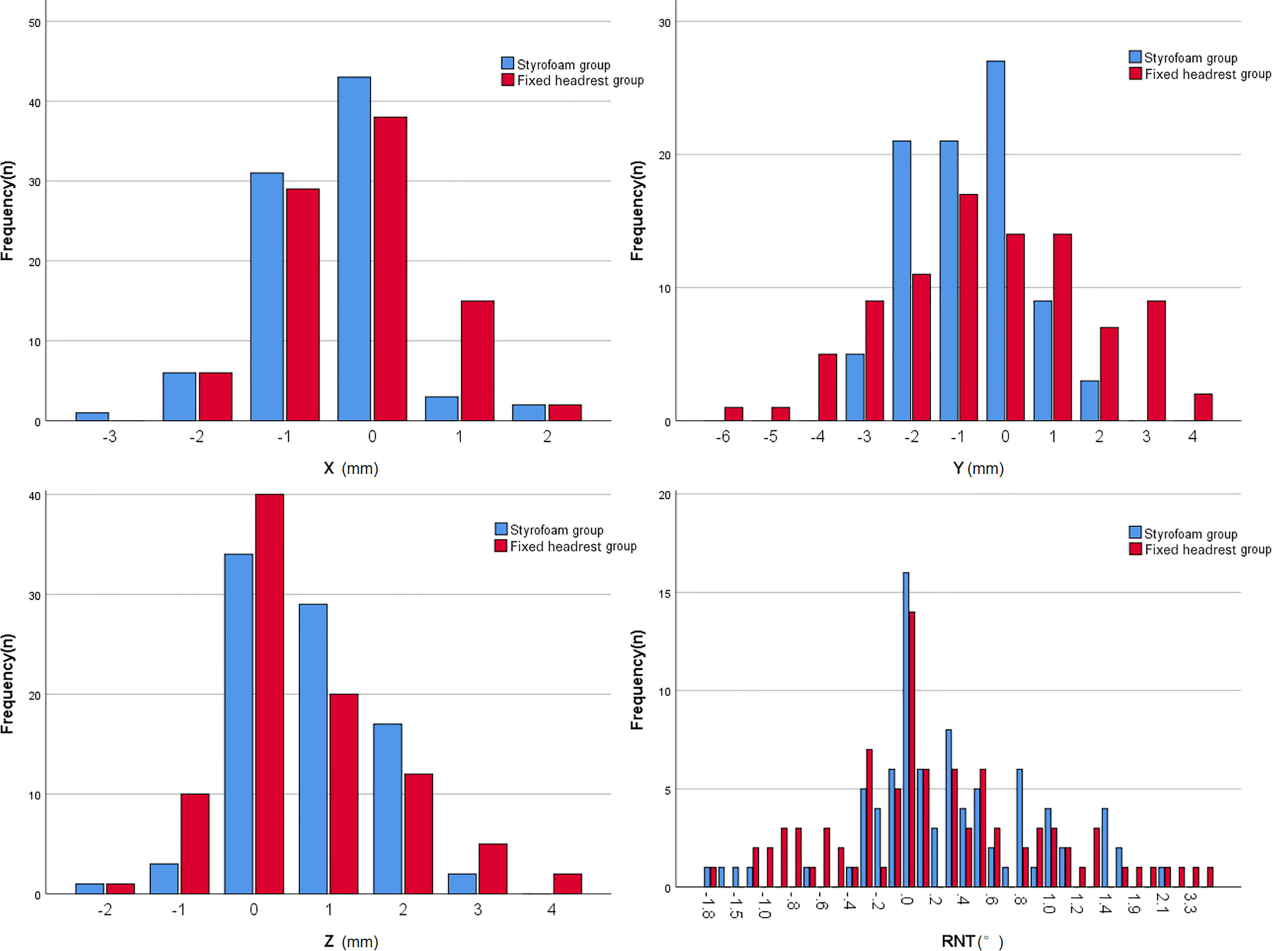
Histogram of the range of set-up error in the two groups of radical patients.

The error ranges in the two groups are shown in **Figure 5**. The errors in the X, Y, Z, and RTN axis directions were 0.5 (0, 1) mm, 1.0 (0, 2) mm, 1.0 (0, 1) mm, and 0.3 (0.1, 0.8)°, respectively, for the foam gel group and 1.0 (0, 1) mm, 1.5 (1, 3) mm, 1.0 (0, 1) mm, and 0.5 (0.1, 0.9)°, respectively, for the headrest group. The positional errors in the Y direction were significantly different (Z = 0.37, P < 0.001) and not significantly different in the X,Z and RNT directions (P > 0.05). The detailed data are shown in **Table 4**.

**Table 4:** Comparison of set-up errors between two groups of patients undergoing radical radiotherapy.

Group	Number of CBCT	Setup errors(mm)			
		X	Y	Z	RTN(°)
Styrofoam group	86	0.5(0,1)	1(0,2)	1(0,1)	0.3(0.1,0.8)
Fixed headrest group	90	1(0,1)	1.5(1,3)	1(0,1)	0.5(0.1,0.9)
Z value		0.770	3.420	0.370	1.454
P		0.441	<0.001	0.709	0.146

### 3.5 Target area expansion boundary

The target area expansion boundaries of the foam group were 1.77 mm, 2.45 mm, and 2.47 mm in the X, Y, and Z directions, respectively, while those of the headrest group were 2.03 mm, 3.88 mm, and 2.57 mm, respectively. The overall size of the foam group was smaller than that of the headrest group.

### 3.6 Subjective comfort satisfaction

**Figure 6:**
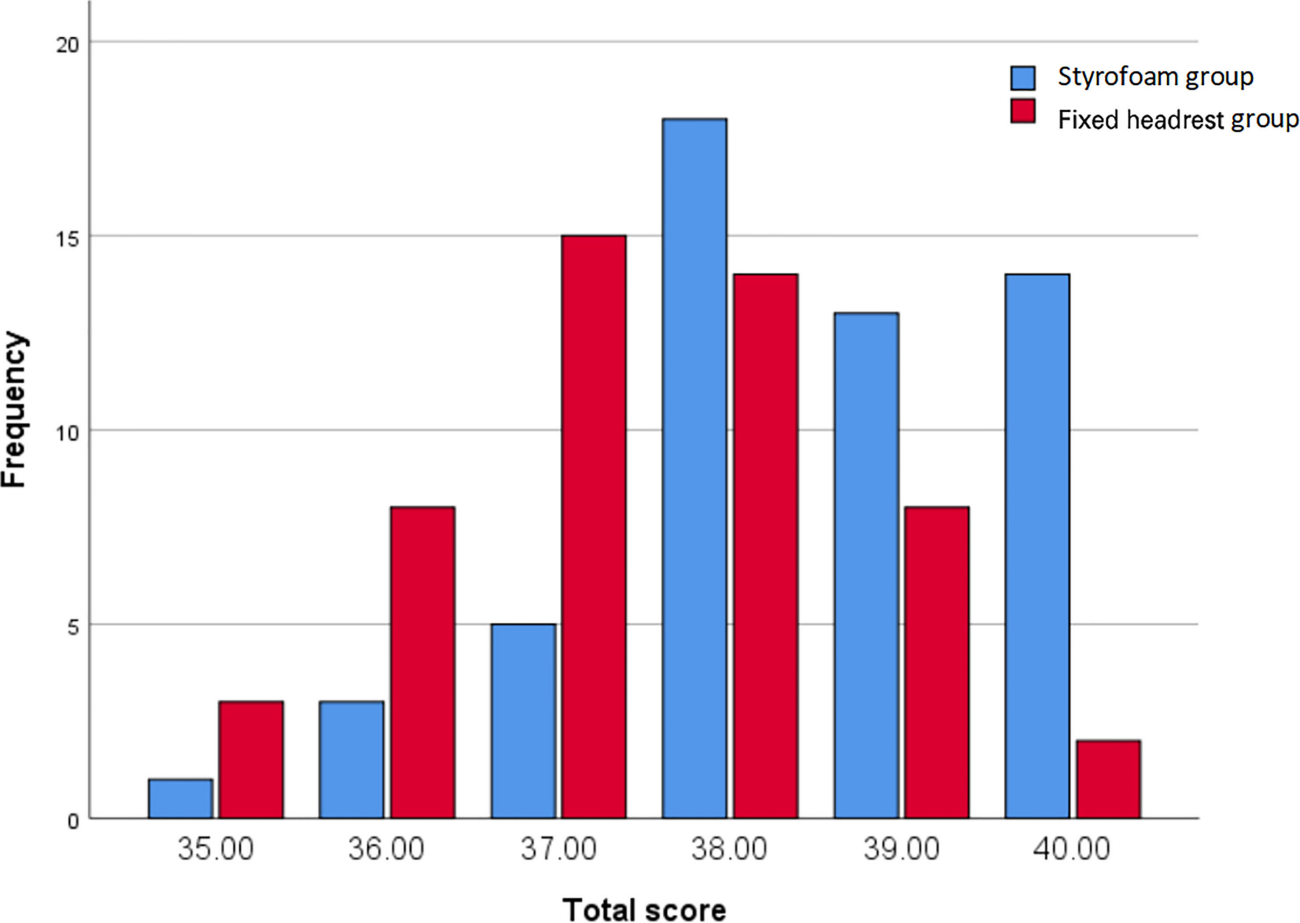
Comfort scores.

The subjective comfort satisfaction scores of the two groups are shown in **Figure 6**. The subjective comfort satisfaction scores of the foam glue group, and headrest group were 38.50 ± 1.24 points and 37.44 ± 1.23 points, respectively. The overall mean of subjective comfort satisfaction score was significantly different between the two groups (difference 1.06, 95% CI, 0.88-1.54, P < 0.001).

### 3.7 Correlation analysis

**Figure 7:**
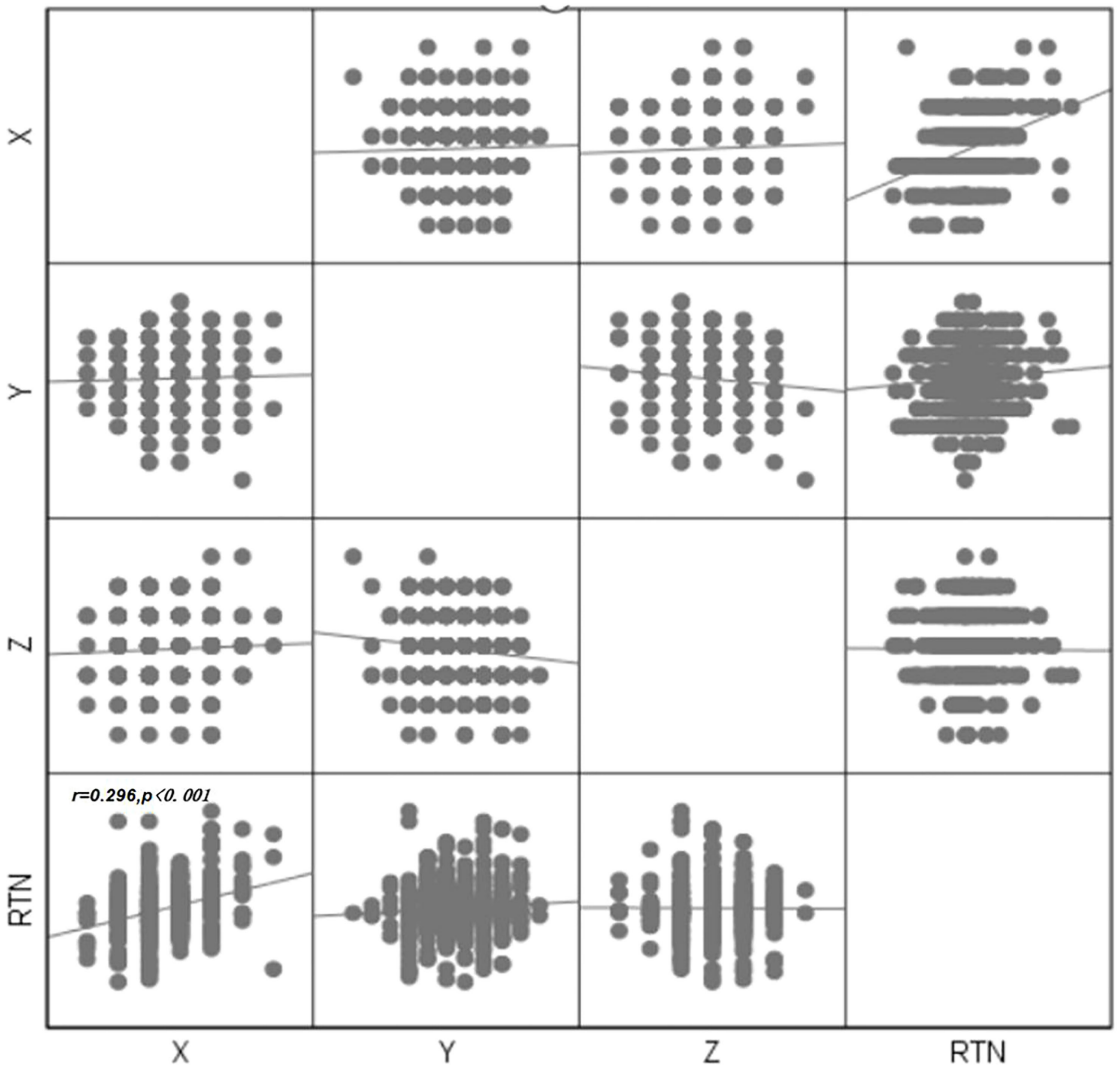
Scatter plot between errors in each direction.

Pearson correlation coefficients and Spearman rank correlation coefficients were evaluated between movements along each pair of translational axes and with rotational axes using each fixation technique. The absolute values of the Pearson correlation coefficient and Spearman rank correlation coefficient were within 0.3 (maximum r=0.296, p=0.000 between X and RTN), indicating that there was no strong correlation between any pair of translational motion variables or with rotational motion variables in the two groups (**Figure 7**).

## 4 Discussion

In this study, CBCT data were collected under two fixation techniques and analyzed to compare the positional accuracy of the styrofoam combined with head model and the fixed headrest combined with head model in intracranial tumor radiation therapy. The styrofoam fixation technique could better reduce the positional error of patients during treatment and improve the positional efficiency and comfort than the fixed headrest fixation technique. Therefore, styrofoam fixation technique of styrofoam combined with head mask is suitable for patients undergoing intracranial tumor radiotherapy.

There were only five fixed sizes of the headrest, which could not be adapted to all patients’ neck curvature, despite the use of a fixed headrest combined with a head mask, a gap between the patient and the headrest may still lead to uncertainty in the patient’s position and affects the patient’s comfort. The styrofoam fixation technique combined with the head mask solves the problem of mismatch between the patient’s neck curvature and the head restraint and also improves the patient’s comfort (11). The styrofoam positioning technique is widely used in radiotherapy for head and neck tumors because personalized fixation of styrofoam can reduce the positional error of neck radiotherapy. However, only a few studies with small sample sizes have used styrofoam positioning techniques for intracranial tumors. In this study, the styrofoam positioning technique had a significantly smaller set-up error in the Y-direction than the fixed headrest fixation technique (P < 0.001). and the comparison between the two groups in the X, Z, and RTN directions was not statistically significant, but according to the histogram only (**Figure 3**),the set-up error with the styrofoam fixation technique was significantly smaller than that with the fixed headrest fixation technique. Furthermore, the positional errors in surgical and non-surgical patients were significantly better in the y-axis of the fixed headrest technique than in styrofoam fixation techniques (**Tables 3**, **4**). The surgical patients with the styrofoam fixation technique outperformed the fixed headrest group in the x-direction, unfortunately not reaching statistical significance (P=0.087). These results further indicate that the styrofoam fixation technique is more useful in postoperative patients than fixed headrest fixation. Notably, according to the histogram (**Figures 4**, **5**), the fixed head-restraint fixation technique is more likely to show data of ≥3 mm (repositioning is required for >3 mm).Furthermore, both the first treatment re-positioning correction rate and the total treatment course re-positioning correction rate were higher for the fixed head-restraint fixation technique than for the styrofoam fixation technique, indicating that styrofoam fixation has better repeatability. The styrofoam group had significantly lower RTN values >2° than the fixed headrest group. Previous studies have shown that a rotation angle > 2° can affect the planned dose distribution (15). Therefore, the position should be readjusted if the set-up error of RTN > 2° since a linear accelerator is not equipped with a six-degrees-of-freedom treatment couch. Furthermore, the fixed headrest is not limited in all directions and can only be fixed by the head mask. The fixed headrest is usually positioned in such a way that the head mask does not completely fit all the patient’s facial skin, taking into account the patient’s comfort and tolerance. However, leaving a gap to ensure comfort may also cause greater positional errors. Nonetheless, an individualized foam fixation technique can wrap the patient’s head and neck, and the fixation of the cephalic mask ensures patient comfort and reduces positioning errors. Also, postoperative patients need more comfortable and individualized fixation due to the discomfort of the surgical site. The styrofoam limits the patient’s head at the vertex, explaining its advantage in the Y-direction.

Lin, CG et al. (11) found that the styrofoam fixation technique can reduce the translation error in nasopharyngeal carcinoma radiotherapy (studied 77 patients with nasopharyngeal carcinoma). They also obtained external expansion boundaries of the target area in the nasopharyngeal region of 2.0 mm, 2.3 mm and 1.6 mm in the X, Y, and Z directions, respectively. Moreover, a smaller external expansion boundaries in the Z direction than we found. Liu, MZ et al. (16) also evaluated the feasibility of open-hole masks combined with styrofoam fixation technique in brain tumors of 14 cases and obtained external expansion boundaries of 2.55, 2.96, and 2.72 mm in the X, Y, and Z directions,21 cases of masks combined with styrofoam fixation obtained corresponding external expansion boundaries of 3.63, 3.69 and 2.97 mm. In this study, Styrofoam fixation obtained external expansion boundaries of 1.77mm and 2.45mm in X and Y directions, respectively, which is smaller than them. Li, TT et al. (17) fixated 19 patients with intracranial metastases using an open head-neck-shoulder mask and fixed headrest and showed similar results in the X and Y directions compared with our fixed headrest group. However, their external expansion boundaries were larger in the Z direction than ours. Overall, our fixation techniques meet the clinical requirements. Han C et al. (18) studied a new positioning technique with better results than the conventional head positioning technique, and also suggested that shortening the duration of radiotherapy can reduce the patient’s intra-fraction motion during radiotherapy and reduce the dose deviation caused by the radiotherapy activity. In this paper, the styrofoam group had shorter positioning duration (32.71 ± 5.21 seconds) and smaller positioning errors than the fixed headrest group (46.57 ± 6.68 seconds). Kang CL et al. (19) also found that intra-fraction motion increases with increasing treatment duration, especially in the x-axis direction. Also, Kang CL et al. could not improve fixation accuracy by adding vacuum pads to the conventional headrest plus mask fixation technique, indicating that a comfortable fixation device is important during radiotherapy. In this study, the subjective comfort satisfaction values of patients in the styrofoam group and fixed headrest group were 88.89% and 70.00%, respectively (P=0.017).

Also, a moderate correlation was found between X orientation and RTN (r=0.29), which was not mentioned in previous studies. The correlation between X and RTN was also consistent with clinical practice, where an increase in RTN increased the X-direction error. A correlation between the Y direction and Z direction was also observed in the unpublished study of head and neck tumors. The lack of correlation in this direction was due to the relative fixation of the skull, and the fact that patients lying too far up or too far down had little effect on the anterior-posterior orientation. The correlation study can be used to analyze the causes of positional errors for appropriate measures.

However, this study has some limitations. First, the set-up error was not analyzed in six dimensions due to the limitations of the conditions. This results in a situation where the set-up error data is less than 3 mm in all three directions, but the physician observes the image and believes that the patient has a rotation that causes the target area to shift, requiring the radiation therapist to re-position the patient. This rotation error data cannot be evaluated. Second, it could not be guaranteed that the patient was positioned by the same radiation therapist during the whole treatment period due to some objective reasons and thus the patient’s status at the time of treatment could not be assessed promptly, and thus may affect experimental data. Third, the effect of treatment duration on the error was not assessed, and thus a follow-up study is needed. Zhang, Bai et al. (20, 21)in our department improved the positional accuracy in X, Y, and Z directions by adding forehead/nasal root auxiliary marker points in the conventional head-rest fixation technique. In this study, this method was not used to compare the accuracy of the two fixation devices. Therefore, the combination of the styrofoam fixation technique and the forehead/nasal root auxiliary marker points can guide future research. This study provides the direction for the next research, prospective experiments, the use of a six-degrees-of-freedom couch, and the effect of treatment duration to make our experimental data more accurate, thus providing a more reliable basis for future clinical radiotherapy.

## 5 Conclusion

The styrofoam plus head thermoplastic mask fixation technology has a higher positioning accuracy, faster positioning efficiency, and better comfort in intracranial tumor radiotherapy than fixed headrest plus head thermoplastic mask fixation. Therefore, styrofoam plus head thermoplastic mask fixation technology can be used in intracranial tumor radiotherapy localization.

## Data availability statement

The raw data supporting the conclusions of this article will be made available by the authors, without undue reservation.

## Ethics statement

The studies involving human participants were reviewed and approved by Medical Ethics Committee of the First Affiliated Hospital of the Air Force Medical University. The patients/participants provided their written informed consent to participate in this study.

## Author contributions

BL, FB, LZ, XY, and LX are the members of team. The study was designed by BL, and FB. BL and FB drafted the initial manuscript. LZ reviewed the manuscript and approved the final version. All authors participated in collecting data. All authors contributed to the article and approved the submitted version.
